# Medorrhinum

**Published:** 1892-11

**Authors:** George H. Clark

**Affiliations:** Germantown, Pa.


### BUREAU OF MATERIA MEDICA.

J. R. HAYNES, M. D., *Chairman.*

---

### MEDORRHINUM.

GEORGE H. CLARK, M. D., GERMANTOWN, PA.

To what place shall we assign Medorrhinum? Is it destined to have the same relation to sycosis that Psorinum has to psora? Shall we be able to use it as an intercurrent in sycotic affections, to give impetus to other remedies?

These questions have come to me after using this remedy in several cases, but I am not yet able to give satisfactory answers.

That the proving of the Medorrhinum we now have is of value can be readily seen by a glance at its pathogenesis, but whether it is to be the remedy *par excellence* for sycosis is to me an unsolved problem. That it is of effect in inherited sycosis I have found out by giving it to a number, but I have been disappointed in its apparently doing nothing, after having done much to lead me to believe that it would permanently cure. As an illustration, a woman, æt. forty-eight years, gave me the following: "Indisposed to work or make mental effort; desire for rest and dread of change; noise, confusion, disorderliness of surroundings distressing; depression and much anxiety, especially after sleep; irritable if the room is not light enough to make everything distinct; craving for stimulants, but cannot endure the effects, for they cause a wild, crazy feeling in brain after the first sleep; same effect from using eyes.

"Shooting pains in head, sometimes in ears. Hearing very acute, but dull of comprehension at times. Aching in back of head and feeling of stiffness and drawing back at top of spine; this always produced by use of eyes. Top of spine as if too weak to support head. Rush of blood to head and face from using eyes, or making any effort, or from any emotion.

"Eyes feel many times as though grains of sand were under lids.

" Feeling of constriction and aching in throat and jaws, when tired.

" When tired, vacant feeling in head ; inability to think continuously, or talk, or to listen to talking.

" Memory very poor, impressions erased, when weary, almost as soon as made.

" Appetite poor, can digest but a limited amount of food.

" Feeling of nausea from using eyes, or when weary. After eating heartily, feeling as though stomach pressed upon a sore spot at left side. Bearing-down sensation in bowels and uterus upon standing or walking, and after bowels are moved. Feeling of want of power in rectum—massing of fecal matter at times.

" According to an old-school doctor, there are ulceration and inflammation of the os uteri, and intra-uterine inflammation of long standing. There is a discharge of a white-of-egg-like substance. Enlargement of uterus. Burning, drawing kind of sensation in uterus after driving, standing on feet, or weariness. Almost constant desire to urinate ; if there be any delay a dreadful nervous feeling, and sometimes burning, sometimes chilly all over body. Urine dark and scanty. Aching in pelvis from leaning over, or if back is not supported.

" Right arm gives out from little use. Lower limbs very weak, especially when first using them after rest."

This from the written description. Questioning as to the mental state, I added : Constant state of anguish. Always a feeling of impending danger, but knows not what. There is no cause for such feelings, as she is not obliged to do anything when disinclined, as she is so situated that nothing need cause her trouble.

She has been an invalid for years. That is, the above symptoms have been almost constant for twenty years.

She has applied to some of the best oculists of the country for relief from the eye symptoms ; but in vain. She has numerous pairs of spectacles, but none afford relief.

To me she presented a perfect picture of sycosis, and I went to work to find a remedy. After study I could find no remedy to cover the entire symptoms, and I then turned to Medorrhinum, as found in *Guiding Symptoms*. After comparing her symptoms I gave her one dose of that remedy.

In a few days she reported that she felt better than for years. For a number of weeks this improvement continued. She was able to go about more than for some time previously, and enjoyed freedom from the most distressing symptoms. But presently there came a standstill. She seemed not to improve. I again compared symptoms and found nothing better than Medorrhinum. Another dose was given, to be again followed by relief, but for a short period only. After waiting, another dose of Med. was given, but it seemed of no effect. She then went away on a coaching tour for a few weeks and returned feeling much better.

In a few weeks more her old symptoms again distressed her, and she was given another dose.

Shortly after this she left for California, and the latest that I have heard is to the effect that she is not much better than when she left.

Another case, undoubtedly inherited sycosis, gave these symptoms: Usually depressed; easily tired; disinclined to any exertion, and yet often unable to keep still; forgetful; unable to think connectedly; mind wanders from subject, even in reading; cannot think at all if hurried.

Always wakens tired in the morning; hates to do anything that must be done, even nice things; gets nervous and excited about riding and driving as soon as the time is fixed.

Sometimes cannot raise lid of right eye after being asleep; cough when tired or nervous.

Aching in right shoulder and arm; dull, sore pain, and soreness and stiffness of arm when moved. Hurts most to move it back or straight out from side. Worse at night or from any pressure on back of shoulder that pushes shoulder forward. Weight of bedclothes on arm hurts it. Aching at times extends to fingers, and the fingers swell when used.

Feet always wet with perspiration. Hands cold. Nails dry and brittle.

Medorrhinum, one dose, was given on November 8th, 1891. The symptoms gradually disappeared and have not returned up to the present.

The question that now arises regarding the first case is: Will Medor. eventually effect a cure, or will other remedies be required? The cases are given for the purpose of eliciting what others have done with this remedy.

### Discussion.

Dr. Close—I have had a case very recently under treatment that might be called membranous dysmenorrhœa. I can give only two or three symptoms from memory. Menstruation was accompanied by terrible pains of a grinding character. Flow scanty, and on the second day there was a passage of a firmly organized membrane. The mental symptom was a constant feeling as if something was behind her. She could not get away from that sensation. It was of nothing definite, simply something terrible behind her, peering over her shoulder, causing her to look around anxiously. Under Medorrhinum I found many of her symptoms, including the peculiar mental symptom, and gave it to her. The next period was without pain and without membranes. The second period had slight pain and passage of small pieces of membrane. Repeated the remedy and the third period was painless again. The fourth has not yet come about.

Dr. Baylies—In a case of erratic rheumatism, the patient, a nervous and anxious man, had a crusty eruption on the upper lip, sometimes dry and sometimes secreting a thin, purulent fluid, like gonorrhœal fluid. Medorrhinum seemed to cover his symptoms, both the rheumatic and the physical and mental depression. I gave it in the 1,000th potency. It appeared to afford rapid but temporary relief to the rheumatic pains, nothing more; and was repeated again and again with the same transient result. Thuja was more effectual, and nearly cured him; but there is still a tendency to recurrence after several months' treatment.

Dr. J. H. Allen—I reported a case of asthma cured by Medorrhinum at the Indiana State Society meeting, 1890. The peculiar symptoms were the mental condition and leucorrhœa of a fishy odor. She had also a cough which kept her sitting up most of the night; the expectoration was yellow. It was cured with one dose of Medorrhinum.

Dr. Clark—To me Medorrhinum has been a disappointment. I have not seen the effects I expected from its use. I have the impression that all the nosodes which we prove should be taken, not from one case only, but from several. Then we should be sure to have a perfect specimen of morbose matter.

